# The ontogeny of synovial tissue macrophages

**DOI:** 10.3389/fimmu.2025.1603473

**Published:** 2025-05-20

**Authors:** Joseph Hutton, Wenrui Sun, Tetsuo Hasegawa

**Affiliations:** ^1^ Cambridge University Hospitals NHS Foundation Trust, Cambridge, United Kingdom; ^2^ Department of Pathology, University of Cambridge, Cambridge, United Kingdom; ^3^ Molecular Immunity Unit, Department of Medicine, University of Cambridge, Medical Research Council Laboratory of Molecular Biology, Cambridge, United Kingdom

**Keywords:** synovium, ontogeny, macrophage, inflammation, osteoclast

## Abstract

Macrophages are essential components of all body tissues, including the synovium. Tissue macrophages originate either from embryonically seeded “primitive” macrophages or from bone marrow-derived monocytes. In adults, both sources contribute to macrophage populations, with their relative proportions varying across tissues and between steady-state and inflammation. Macrophages are highly responsive to microenvironmental and signalling cues, which significantly influence their function within tissues. This article reviews the current understanding of synovial tissue macrophage ontogeny in health and disease, highlighting knowledge gaps and potential avenues for future research.

## Introduction

Macrophages are present in every body tissue and are fundamental immune cells in both tissue homeostasis and inflammation. They perform diverse tissue-specific functions, protecting against infections and other noxious insults (1, 2). However, they can also contribute to disease pathophysiology, including inflammatory arthritis (IA) (3). The function of tissue-resident macrophages is shaped by their microenvironment, signalling cues, cross-talk with other cell populations, and ontogeny (3–5). Here, we provide a brief overview of macrophage roles in synovial tissue homeostasis and immunity, with a particular focus on their developmental origins and relevance to health and disease.

Macrophages are indispensable immune cells found in every organ and are among the first immune cells to form during embryogenesis (6). They share core functions essential for tissue homeostasis and surveillance, including clearing damaged cells, foreign bodies, and pathogens (1, 5, 6). Additionally, macrophages help maintain tissue integrity by shielding these elements from recognition by other immune cells (1). Beyond their role in immune defence, macrophages contribute to tissue development and repair, supporting vascular integrity, angiogenesis and organogenesis (1, 5, 6). Many macrophages also have specialised functions unique to their resident tissues: for example, microglia in the brain prune neurons, alveolar macrophages recycle lung surfactant, and osteoclasts resorb bone (1, 7, 8).

These tissue-specific roles extend to the synovium, where synovial tissue macrophages (STMs) are crucial for joint health and function (3, 9). STMs have long been recognised as key players in inflammatory arthritis, producing pro-inflammatory mediators such as tumour necrosis factor (TNF), and actively contributing to the maintenance of remission (3, 9). Over the past decade, advances in fate-mapping technologies have significantly expanded our understanding of macrophage ontogeny (1, 2). Tissue macrophages originate from embryonically seeded “primitive” macrophages and are maintained through tissue-specific replenishment by monocyte-derived macrophages in steady-state conditions (1, 2, 10). The balance of macrophage origins shifts during inflammation, with a greater proportion of cells arising from circulating monocytes in disease states (2, 3, 6). This shift may influence their function within the tissue.

## Macrophage ontogeny

### Embryonic origins of macrophages

During gestation, haematopoiesis occurs in successive waves across different sites in the developing foetus (1, 11, 12). Starting at embryonic day 8.5 (E8.5), the yolk sac generates early erythromyeloid progenitors (EMPs) which colonise foetal tissues from E9 onwards (1, 11, 12). These progenitors differentiate directly into tissue-resident macrophages without passing through a monocyte intermediate (1, 11, 12). Known as “primitive” macrophages, they lack *HLF* and major histocompatibility complex (MHC)-II markers, both of which are present on monocyte-derived macrophages originating from haematopoietic stem cells (HSCs) (10). However, MHC-II expression alone is not a reliable distinguisher of ontogeny. This is as studies have demonstrated that bone fide primitive macrophages (Hofbauer cells in the placenta, microglia) slowly acquire MHC-II expression in tissue (10).

Other foetal structures such as the placenta also produce *de novo* placenta-associated erythromyeloid progenitors (PEMP) that form placental macrophages prior to foetal blood flow and connection of the vasculature (10). As gestation progresses, the foetus transitions to definitive haematopoieisis (10, 13–15). Briefly, the aorta-gonado-mesonephros (AGM) generates HSC which seed the foetal liver (16). The foetal liver acts as the primary site of haematopoiesis with rapid expansion of HSC which then seed the foetal bone marrow (17). In humans, AGM production of HSC begins at Carnegie stage 13, approximately 27 days post-conception (18, 19). In humans, the foetal bone marrow becomes an active site of haematopoiesis much earlier than in mice by the end of the first trimester 10–11 weeks post conception (20). HSC give rise to monocytes, which can then form monocyte-derived tissue macrophages (21, 22). Notably, even primitive tissue macrophages arising from the earliest stages of embryogenesis can self-maintain and proliferate over the course of an organisms lifespan (1, 21, 23). Therefore, primitive macrophages can be found even in adulthood. The degree to which these cells persist in adult humans is unclear given our long life-spans, non-sterile environments, and lack of tools to readily explore ontogeny in humans (1).

### Haematopoietic and monocyte-derived macrophages

Definitive haematopoiesis persists throughout childhood and adulthood. This process, driven by haematopoietic stem cells (HSCs), follows a complex hierarchy of progenitors with progressively restricted lineage potential (16, 24). Ultimately, one such output are circulating blood monocytes, which can enter tissues and adopt various fates, including differentiation into tissue-resident macrophages (1, 6, 22, 25).

The contribution of monocyte-derived macrophages to tissue macrophage pools varies depending on several factors. These include the organism’s age, the tissue’s immune privilege, the presence of available niches, and prior inflammatory insults to the tissue (1). Some tissue-resident macrophage populations may even have individual cells of mixed primitive and definitive origin (7). This is exemplified by multinucleated osteoclasts, where monocyte-derived cells progressively fuse with tissue resident foetal-derived osteoclasts to form multinucleated syncytia (7). This process may be relevant to inflammatory and erosive joint diseases, as macrophages predisposed to osteoclastogenesis in the inflamed synovium—known as arthritis-associated osteoclastogenic macrophages (AtoMs)—have been identified in these conditions (26).

**Figure 1:**
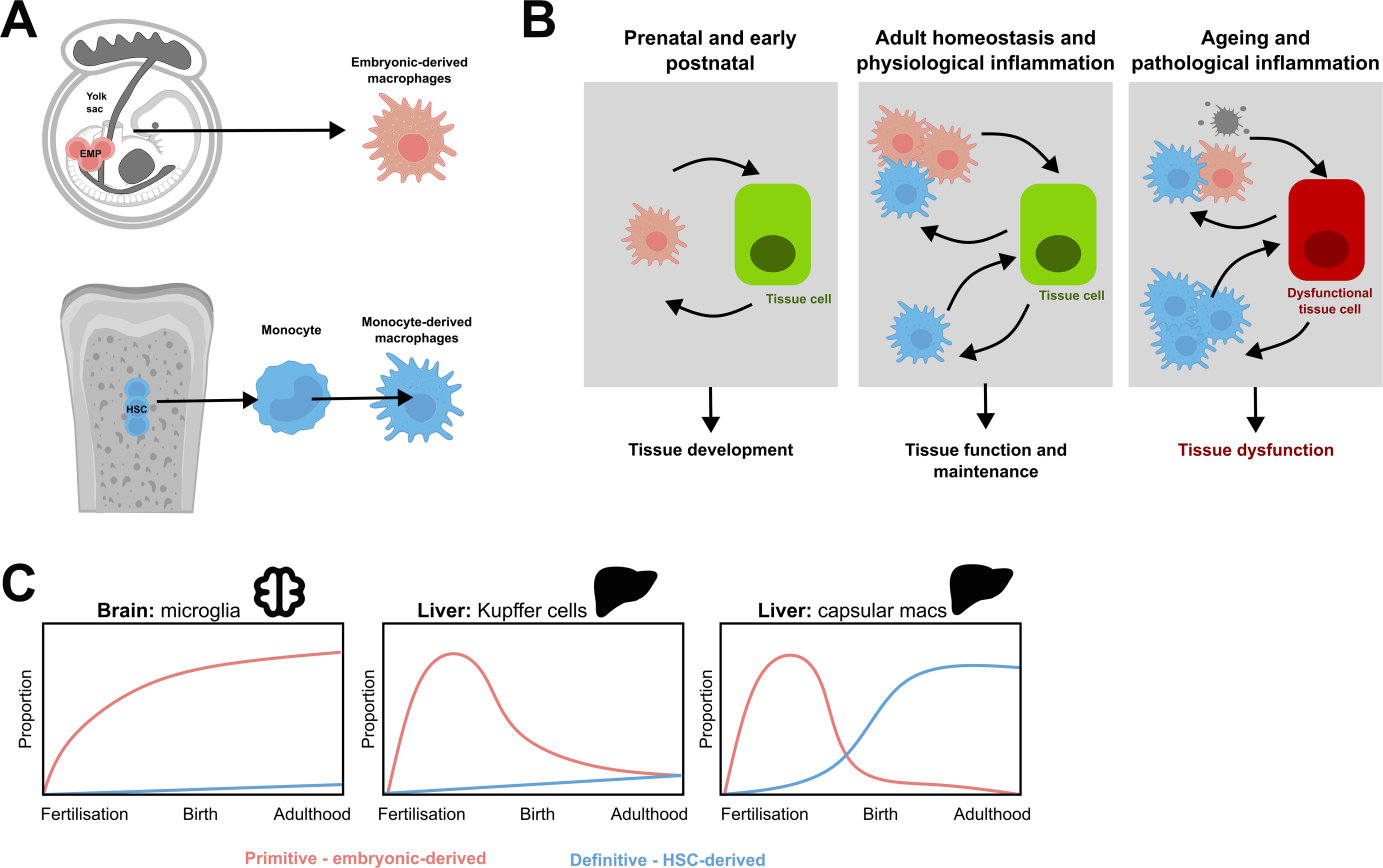
Distinct ontogenies of resident tissue macrophage populations. **(A)** During embryogenesis, yolk sac erythromyeloid progenitors (EMPs) give rise to cells that differentiate into long-lived resident tissue macrophages. As gestation progresses, the embryo switches from primitive to definitive haematopoiesis, and the bone marrow eventually takes over as the main source of haematopoiesis. This produces HSC which produce monocytes which can migrate into tissues and form monocyte derived macrophages. **(B)** Both primitive macrophages and monocyte-derived macrophages contribute to tissue biology and exhibit cell-cell communication with tissue cells. During embryogenesis, prenatal, and postnatal life, tissue development is reliant on primitive macrophages. After birth, homeostasis distinct macrophage subpopulations may have different functions and cell-cell communications with healthy tissue cells to support and maintain tissue function. On ageing or pathological inflammation, this balance is disrupted. This can trigger apoptosis in resident macrophages, increased recruitment of monocyte-derived macrophages, directly damage other cells, and contribute to the dysfunction of the tissue. Modified from Mass et al, 2023 (1). **(C)** Schematic demonstrating the differing contributions of primitive and definitive haematopoieisis to tissue-resident macrophage populations in murine organs. Modified from Ginhoux and Guilliams, 2016 (2) and Thomas et al, 2023 (10).

Recent findings suggest that monocyte-derived macrophages may have more complex origins than previously thought. In mice, monocytes can arise from distinct HSC-derived progenitors, including granulocyte-macrophage progenitors (GMPs) and macrophage-dendritic cell progenitors (MDPs) (27). While both GMP- and MDP-derived monocytes populate the gastrointestinal tract equally, they exhibit distinct seeding patterns in other tissues, such as the lung and brain (27) (**Figure 1**). Although robust markers distinguishing these lineages have yet to be identified in humans, it is likely that similar developmental pathways and tissue biases exist in human macrophage populations.

Another factor influencing the contribution of monocyte-derived macrophages to tissues is their likelihood of engraftment. It is assumed that all monocytes entering a tissue have an equal chance of occupying an empty niche, but this is not necessarily the case. This is evidenced from these recent findings of GMP- and MDP-origin monocytes in mice (27). Similarly, there is significant evidence that suggests circulating leukocytes in inflammatory arthritis have defects in the processes that regulate their entry and exit into tissues (28–30). This implies that specific monocyte populations may be primed for migration into particular tissues, influencing their ability to engraft and contribute to the resident macrophage pool.

### Does origin matter?

It remains unclear whether macrophage ontogeny significantly influences their function within tissues. Previous studies have explored the similarities between engrafted monocyte-derived macrophages and primitive macrophages. For instance, Scott et al, 2016 reported that monocyte-derived Kupffer cells exhibited an almost identical transcriptomic profile to their embryonically derived counterparts (31). However, other studies have identified over 2,000 differentially expressed genes between monocyte-derived microglia and primitive microglia, suggesting that ontogeny may indeed play a role (32).

Recent findings further complicate this question. Some studies suggest that monocytes can seed specific brain regions in healthy aging, closely resembling embryonically derived microglia at the transcriptional level (33). Additionally, the discovery of GMP- and MDP-derived monocytes with distinct tissue tropisms in mice supports the idea that origin may matter, potentially in a tissue-specific manner (27). Strong evidence for this exists in murine models of rheumatoid arthritis (RA), where AtoMs which originate from blood monocytes give rise to osteoclasts that bind to and resorb bone more indiscriminately than healthy osteoclasts (26, 34).

Before 2010, studies rarely differentiated between primitive and monocyte-derived macrophages, leaving their respective roles within tissues largely unexplored (1). Furthermore, much of the research has relied on murine fate-tracking models or pathological conditions such as post-bone marrow transplantation in humans (35, 36), where cellular behaviour may not fully reflect normal homeostasis.

## Synovial tissue macrophages and the joint microenvironment

### Normal synovial structure

**Figure 2:**
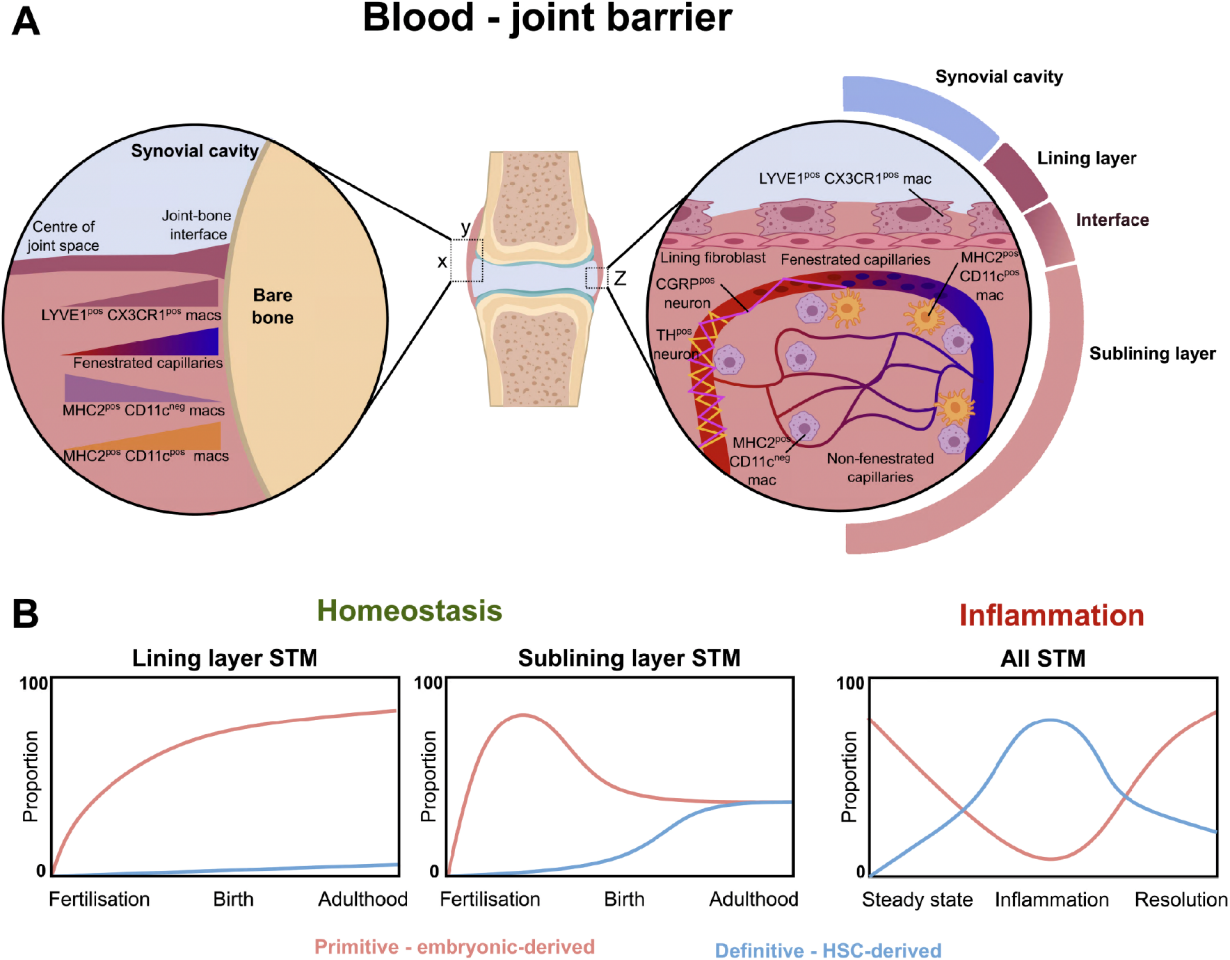
The blood-joint barrier and the ontogeny of synovial tissue macrophages. **(A)** Diagram demonstrating the spatial distribution of synovial tissue macrophages, fenestrated endothelium, and neurons. Left zoom-in demonstrates changing density of different macrophage populations and fenestrated endothelium across XY axis of joint. Right zoom-in demonstrates Z axis of joint, with lining layer formed by MHC-II^neg^ LYVE1^pos^ CX3CR1^pos^ macrophages, with supporting sublayer of lining fibroblasts. MHC2^pos^ CD11c^neg^ and MH2^pos^ CD11c^pos^ macrophages survey the area around fenestrated capillaries. **(B)** Primitive macrophages (F4/80^pos^, CD11b^neg^) contribute to the majority of lining and sublining layer STM during gestation. After birth, there are increasing contributions of monocyte-derived CD11b^pos^ macrophages to the sublining layer (49). Similar postnatal findings demonstrated using Ms4a3-tdTomato fate-tracker model in Hasegawa et al, 2024 (39). During inflammation in the collagen induced arthritis model (49), embryonic-derived macrophages decline over the course of inflammation, with eventual recovery during resolution. Conversely, the proportion of monocyte-derived macrophages increases in the synovium over the course of inflammation, before waning as the resolution phase progresses.

The synovial membrane is a specialised soft tissue that lines the inner surface of the fibrous capsule surrounding synovial joints. It plays a crucial role in the normal function of joints, bursae, and tendon sheaths (37, 38). Its primary functions include lubricating the joint to minimise friction, providing metabolic and nutritional support to the synovial cavity, particularly to the avascular cartilage (37, 38). It also serves as a “blood-joint barrier” to help defend joint tissue (39) (**Figure 2A**).

The synovial membrane consists of diverse specialised cell populations and subpopulations, each occupying distinct tissue niches and performing unique homeostatic functions (37). Structurally, it is composed of a thin lining layer adjacent to the joint cavity and a larger sublining layer (36). In humans, synovial joint development begins around 5–6 weeks post-conception, with cavity formation occurring between 7–8 weeks, depending on the specific joint (40).

### Synovial tissue macrophage subpopulations

Macrophages are essential components of both the lining and sublining layers of the synovium in mice and humans. In humans, multiple subpopulations of STMs have been identified, though a full discussion of these populations in both species has been comprehensively reviewed elsewhere (41). Here, we focus on human STMs. These are important cells both for normal synovial function, as well as in conditions like rheumatoid arthritis. In RA, macrophage abundance correlates with joint damage, disease activity, and are principal producers of pro-inflammatory markers like TNF and IL6 (41). Many of these pro-inflammatory macrophages are believed to be derived from tissue-infiltrating monocytes (41). STM can be polarised into a pro-inflammatory state or tissue-reparative states. In RA, there is an imbalance, with more pro-inflammatory phenotypes than tissue-reparative states within the joint, leading to secretion of pro-inflammatory cytokines and osteoclast activation, as well as complex signalling pathways that drive further macrophage polarisation towards inflammatory phenotypes (42). Inflammatory macrophages also stimulate fibroblasts to produce IL-6, prostaglandins and matrix-metalloproteinases (42).

Alivernini et al, 2020 (9) describe MerTK^pos^ CD206^pos^ macrophages, which consist of two main subpopulations. One is a TREM2^pos^ CX3CR1^pos^ FOLR2^pos^ macrophage population that forms the lining layer (9) and is thought to be homologous to the Cd64^pos^ MHC-II^neg^ STMs in mice (43). The sublining layer contains LYVE1^pos^ FOLR2^pos^ macrophages (9), though LYVE1 is also expressed by healthy lining layer cells (9, 39), making its discriminatory value uncertain. Both populations include distinct subclusters that reflect different activation states (9, 41). Additionally, synovial tissue contains a substantial number of CLEC10A^pos^ macrophages with a dendritic cell (DC)-like phenotype (9), though recent evidence suggests that at least some of these cells may be true synovial DCs (44). In inflammatory arthritis, all these populations are disrupted, with an influx of MerTK^neg^ CD206^neg^ macrophages, likely representing monocyte-derived macrophages (9, 41). These inflammatory changes and their implications are explored in the following sections.

### The healthy lining layer

In health, the lining layer is 1–3 cells thick and consists of tissue-resident macrophages and fibroblasts. These overlie a fine fibrillar matrix of collagen and laminin fibres that form a basement-like membrane (37, 38). This membrane supports the lining cells and facilitates the controlled flow of cells and molecules between the synovium and the sterile fluid-filled cavity (37). Studies have shown that lining macrophages and their processes are separated by intercellular spaces, and direct continuity between joint cavity and intercellular spaces are observed using electron microscopy (45–47). A study reported that lining layer macrophages are enriched for genes in tight junction and barrier functions, e.g. tight junction protein 1 (TJP1) (43). Additionally, these STM also have high expression of transcripts for scavenging receptors, lipid-binding proteins, and phagosomal components, in keeping with their proposed role in clearing apoptotic cells and bacteria from the joint (9, 41). Although sagittal sections have been used as the gold standard to define the spatial organisation of synovial cells, recent study developed whole mount imaging system to fully capture the complex anatomy of the synovium (39). This novel imaging system revealed the heterogeneous distribution patterns of the lining macrophages across the synovium and intercellular spaces between lining macrophages, especially in the central area of the synovium (39). In mice, these lining macrophages are long-lived and locally renew from joint-resident precursors that are seeded during embryogenesis (43). The ontogeny of their human equivalent is unknown.

### The healthy sublining layer

The sublining layer is a looser collagenous connective tissue network and relatively acellular in health (37, 38). It contains fibroblasts, STMs, nerves, blood and lymphatic vessels. Other cells such as adipocytes, lymphocytes, and mast cells can also be found (37, 38). The sublining layer functions to transport cells, nutrients, oxygen, waste and lymph to and from the synovium (37). The connective tissue matrix also plays a role in regulating stromal cell motility and adhesion, as well as tissue stiffness (48). In health, the majority of sublining STMs are MerTK^pos^, and include the LYVE1^pos^ cells and MerTK^pos^ ID2^pos^ cells (9, 41). MerTK^pos^ ID2^pos^ cells are believed to represent a locally renewing STM due to their high expression of macrophage colony stimulating factor (M-CSF) and ID2 (9, 41). These features are shared with self-renewing HSC, and murine STM precursors (9, 43). Other populations can be found in the sublining layer in humans, including the DC-like CLEC10A^pos^ cells (9).

### Recent advances in understanding of STM and synovial microarchitecture

Recent advancements in technology and imaging techniques have significantly refined our understanding of synovial structure, particularly the localisation of STMs (39). Whole-mount immunofluorescence imaging of the murine knee joint synovium has provided a more detailed view of its complex architecture (39).

One key finding is the presence of fenestrated capillaries (Plvap^+^/PV1^+^) within the synovium, particularly at the periphery of the joint near the synovium-bone interface (39). These capillaries are located just beneath the lining layer, at the lining-sublining interface. STMs exhibit distinct distribution patterns within this microenvironment. MHC-II^neg^ LYVE1^pos^ CX3CR1^pos^ macrophages form the surface lining layer and interface with the underlying sublining layer, MHC-II^pos^ CD11c^pos^ STMs are closely associated with PV1^pos^ fenestrated vessels (39). The majority of the remaining sublining macrophages consist of MHC-II^pos^ CD11c^neg^ STMs. Notably, fenestrated capillaries allow free extravasation of immune complexes, a feature that intriguingly aligns with regions most susceptible to pannus formation in inflammatory arthritis (39).

These capillaries and the surrounding macrophages are also intricately linked with nociceptive neurons, acting as “sentinels” for systemic inflammation (39).

Collectively, these findings reveal that the synovium is structurally more complex than previously thought. Distinct STM populations reinforce permeable areas of joint tissue, playing a critical role in maintaining the blood-joint barrier. This unique organisation may also help explain the synovium’s vulnerability to specific infections, its role in pannus formation, and the strong associations between inflammatory arthritis, pain sensitisation, and fibromyalgia (39).

## The ontogeny of STM in health and disease state

Only in recent years have the necessary tools become available to investigate these questions in depth (1, 2). Currently, these tools are largely limited to murine models, which provide invaluable insights into human biology, though no definitive technology yet exists to explore these mechanisms directly in humans. Various mouse models have been employed to study macrophage ontogeny and function, including CX3CR1-EGFP models, CCR2^neg/neg^ models, bone marrow chimeric models, as well as Flt3^cre^, and CD115^creER^ mice. While it is beyond the scope of this work to fully discuss these, in general, they work on the basis that specific cell types express a particular gene of interest. Cell types expressing these genes, and their progeny are irreversibly labelled with a fluorescent tag, allowing for their ontogeny to be demonstrated when tissues are later sampled. These can be on the basis purely on expression of a target gene, where all cells expressing that gene and their progeny are labelled, or can be induced at a specific time point (e.g. by tamoxifen) to label cells in a particular developmental window. One such example is the recent Ms4A3-tdTomato mouse which allows the unambiguous identification of monocyte-derived cells (22). *Ms4a3* is a gene that is highly and specifically expressed in GMPs, granulocyte progenitors (GPs), and common monocyte progenitors (cMoPs) (22). It is not expressed during primitive haematopoiesis, in tissue-resident macrophages, or dendritic cells. Utilising the *Ms4a3Cre-RosaTdT*omato model therefore permits labelling of cells that express *Ms4a3* during their differentiation, including monocyte-derived macrophages which thereafter constitutively express TdTomato and can be detected via flow cytometry or immunofluorescence (22).

### Embryonic development of STM

Previously, F4/80 and CD11b (*Itgam*) have been used to distinguish between macrophages of embryonic origin and monocyte-derived macrophages arising from definitive haematopoiesis (49). In the synovium, CX3CR1-GFP^pos^ macrophages, used as a proxy marker for STMs, first localise around the developing murine joint at E12.5 (49). At this stage, these cells are small and difficult to distinguish morphologically from other developing joint tissues (49).

F4/80^pos^ cells subsequently appear, with a distinct synovial structure becoming visible by E15.5 (49). The number of F4/80^pos^ macrophages continues to increase throughout the remainder of embryonic development, likely through local proliferation, as evidenced by 65% of F4/80^pos^ STMs co-expressing the cell-cycle marker Ki67 at E16.5 (49). In contrast, CD11b^pos^ bone marrow-derived macrophages do not populate the developing synovium until E18.5 (49).

Between E20.5 and postnatal day 7 (P7), a distinct population F4/80^neg^ CD11b^pos^ STMs emerges within synovial tissue (49). These cells also express other markers associated with definitive haematopoiesis, such as Ly6C. Notably, the total number of embryonic STMs remains unaffected in CCR2-deficient mice (49). Since CCR2 is a key regulator of monocyte egress from the bone marrow, this finding supports the hypothesis that embryonic STMs develop independently of HSCs.

### Steady-state synovium

As mice become older, a heterogenous pattern of STM origin is observed. The number of F4/80^pos^ CD11b^neg^ embryonic macrophages gradually increases, but their proliferation greatly reduces (49). By 8 weeks of age, there is negligible Ki67 expression in these cells. The number of F4/80^neg^ CD11b^pos^ bone-marrow derived macrophages gradually decreases, and a mixed population of F4/80^pos^ CD11b^pos^ cells increases to adulthood (49). Similar findings were found using bone marrow chimeric mice, utilising CD45.1 and CD45.2. Briefly, CD45.1 host mice were irradiated and CD45.2 bone marrow HSC transplanted (49). After 2 months, mice were sacrificed and the synovial compartment analysed via flow cytometry. Over 30% of the total synovial macrophages were from the CD45.2 donor indicating monocyte-origins. However all F4/80^pos^ CD11b^neg^ STM, consistent with embryonic origin were from the CD45.1 recipient, demonstrating the utility of these markers in discriminating murine STM ontogeny. This pattern is consistent with slow replenishment of STM via circulating monocyte-derived cells (49).

Hasegawa et al, 2024 demonstrated that circulating monocyte-derived cells preferentially replenish specific STM subsets (39). Using the Ms4a3-tdTomato mouse model, they found that under steady-state conditions, nearly 50% of MHC-II^pos^ STM were tdTomato^pos^, indicating a bone marrow-derived origin (39). These cells were predominantly located at the periphery of the synovium near the joint-bone interface. In contrast, the majority of lining layer LYVE1^pos^ CX3CR1^pos^ STMs were tdTomato^neg^; consistent with an embryonic origin (39) (**Figure 2B**). This pattern of replacement kinetics appears heterogeneous compared to other organ systems. For instance, brain microglia, liver Kupffer cells, and skin Langerhans cells exhibit minimal monocyte contribution in the steady state (4, 10–12, 31, 39). However, even in these sites, subset specific monocyte replenishment can be observed, with liver capsular macrophages and border-associated macrophages having distinct ontogeny and replenishment characteristics compared to microglia and Kupffer cells (50, 51). Meanwhile, kidney and spleen macrophages undergo slow monocyte-driven replenishment, whereas macrophages in the gut and other skin regions are replaced at a much faster rate (10, 12). Lining macrophages which have their origin from yolk-sac (Ms4a3^neg^) are enriched for TNF stimulation signatures, whereas interstitial CD11c^pos^MHCII^pos^ mononuclear phagocytes which have their origin from both yolk-sac and monocytes are enriched for gene sets associated with M2-like stimuli (IL-4 and IL-13) (39).

### The synovium in pathology

The recent study further investigated STM dynamics by challenging mice with 48-hour intravenous immune-complex exposure (39). By 72 hours, they observed the formation of MHC-II^pos^ macrophage aggregates within the synovium. A similar response was noted following oral challenge with *Salmonella enterica* serovar *Typhimurium*, whereas no such aggregates appeared in urinary tract infection models using *E. coli (* 39 *)*. To track cell origin, immune-complex challenge was combined with intravenous administration of CD45-PE at the start of the experiment, followed by CD45-AF488 injection 24 hours before sacrifice (39). While leukocytes labelled at both time points were detectable in tissues such as the spleen, no labelled cells were found within STM aggregates (39). Additionally, these aggregates were negative for tdTomato, suggesting that they formed through local STM proliferation rather than monocyte recruitment (39).

The impact of inflammatory arthritis has also been studied using the collagen-induced arthritis (CIA) model (49). During disease onset, the population of F4/80^pos^ CD11b^neg^ embryonic macrophages gradually declined, reaching their lowest numbers at peak inflammation (49). However, their numbers slowly recovered as the disease resolved. In contrast, F4/80^neg^ CD11b^pos^ STM progressively increased in synovial tissues over the course of CIA, in keeping with bone marrow-derived inflammatory cell influx (49). Their numbers diminished as the inflammation subsided. Notably, these STM populations exhibited distinct functional phenotypes: embryonic macrophages generally displayed a more reparative, M2-like profile, whereas bone marrow-derived STM were predominantly pro-inflammatory (49).

### Findings for other synovial-relevant macrophages: osteoclasts

Osteoclasts are highly specialised multinucleated giant cells primarily responsible for bone resorption (8, 52–57). *In vitro*, they form through the fusion of monocytes into multinucleated syncytia, a process regulated by M-CSF and RANKL (8, 52–57). While osteoclasts play essential roles in healthy bone turnover and maintaining the haematopoietic niche within bone marrow (1, 7, 8), they contribute to pathological bone resorption and joint damage in IA (26).

During embryogenesis, osteoclasts first appear at E15, originating from primitive macrophages (7, 8, 58). To study both embryonic and bone marrow-derived osteoclasts, CSF1R knockout mice (CSF1R^cre^/TNFRSF11A^fl/fl^) have been used (7). These mice exhibit abnormal bone formation, increased bone density, failed tooth eruption, and deficiencies in both osteoclasts and HSCs (7). Conversely, CSF1R can be abrogated in bone-marrow derived cells only using CSF1R^cre^/Rosa26^LSL-YFP^ mice (7). This results in normal osteoclast numbers and bone phenotype at birth. However, over 22–60 weeks, these mice display increased bone density and reduced HSCs in long bones due to the gradual loss of monocyte-derived osteoclasts (7).

Reporter mouse models targeting tartrate-resistant acid phosphatase (TRAP) have further elucidated osteoclast ontogeny. CSF1R^mer-icre-mer^/Rosa26^LSL-YFP^ mice pulsed with tamoxifen at E8.5 selectively label embryonic macrophages but not HSC-derived cells (7). Briefly, these fate-mapping models contains a Cre recombinase fused to a mutant oestrogen ligand-binding domain (CreERT2) that requires the oestrogen antagonist tamoxifen for activity. Upon tamoxifen injection, targeted cells will start to express the fluorescent reporter, permitting induction of labelling at controlled manner at particular timepoints or developmental stages (59). Such models do feature a number of limitations, including a lag in labelling after exposure, requirements for careful experimental controls, and the need to cross suitable mouse strains to successfully target genes of interest (59). Parabiosis experiments, joining these mice with CSF1R^cre^/Rosa26^LSL-tdTomato^ mice for 4–8 weeks, allowed researchers to track osteoclast nuclei replacement. Embryonic-origin osteoclasts were labelled with yellow-fluorescent protein (YFP), while monocyte-derived osteoclasts were marked with tdTomato. This revealed a gradual incorporation of tdTomato-positive monocyte-derived nuclei into multinucleated osteoclasts, suggesting an ongoing contribution of bone marrow-derived monocytes to osteoclasts (7). These findings have implications for disease states. In both parabiosis and bone marrow chimera experiments using TRAP-tdTomato mice, pathological osteoclasts at the pannus-bone interface in IA were shown to arise from monocyte-derived macrophages, AtoMs, reinforcing their role in joint destruction (26).

### Human STM

Limited research has been conducted in humans due to the lack of reliable tools and proxy markers needed to distinguish between blood-derived and embryonic macrophages. Tu et al (49 *)* investigated RA synovium and found a significantly higher number of CD11b^pos^ STM compared to OA, as observed through immunofluorescence. These CD11b^pos^ cells were predominantly localised around blood vessels (49). Meanwhile, STM positive for EMR1, the human homologue of F4/80, were found outside of blood vessels. These distinct STM populations exhibited similar M1/M2 polarisation phenotypes to those seen in murine models, supporting the hypothesis that STM of differing ontogenies exist within inflamed synovium (49). These findings align with the work of explorations of human STM populations *(* 9, 60, 61 *).* Many of these macrophages populations are likely monocyte-derived based on their transcriptome and expansion in active synovitis (41), although this has not been definitively proven. Finally, imaging studies using technetium-^99m^ labelled monocytes have demonstrated monocyte accumulation in active RA joints (62–64). Although the ultimate fate of these monocytes remains uncertain, it is likely that at least some differentiate into monocyte-derived macrophages within the inflamed synovium. Further work employing human bone marrow transplant recipients (35, 36), pulsed deuterium to label HSC-origin cells (21, 65), or as-yet undiscovered methodologies to reliably discriminate monocyte-derived cells should be employed to explore this hypothesis.

## Conclusions and future directions

In summary multiple subsets of STM exist within the murine and human joint, each with subset-specific roles in homeostasis and disease-state. STM are believed to be embryologically-derived, with subset-specific replenishment of cells from monocyte-derived macrophages across life in steady-state. However, in synovial pathology, both locally-resident STM can proliferate in response to circulating insults, and an influx of monocyte-derived macrophages may be seen. Despite our current knowledge, significant gaps remain. These include the specifics of how embryonic STM are homeostatically maintained and their longevity over the lifespan of humans. Most murine models are specific-pathogen free, whereas humans exist in a dirty environment, with considerably heterogenous microbiota, and free exposure to pathogens. Additionally, our lifespan is considerably longer than that of mice, thus the contribution of monocyte-derived macrophages to the STM pool may be considerably higher. The signals that govern the differentiation of monocytes into a healthy, homeostatic STM vs. damaging pro-inflammatory macrophages still requires elucidation. The cross-talk of different STMs, and their divergent ontogeny with other joint-resident populations such as neurons in musculoskeletal pain is also poorly understood. Finally, the dynamics and specific mechanisms that guide infiltrating monocytes into synovial tissue require further exploration.

## Supplementary material

Supplementary Table 1 A taxonomy of human and mouse STM. *The MerTK+LYVE1+FOLR2^high^ cluster was reported to be localised in the lining layer in both healthy and remission RA (9).

## References

[1] Mass E Nimmerjahn F Kierdorf K Schlitzer A. Tissue-specific macrophages: how they develop and choreograph tissue biology. Nat Rev Immunol. (2023) 23:563–79. doi: 10.1038/s41577-023-00848-y PMC10017071 36922638

[2] Ginhoux F Guilliams M. Tissue-resident macrophage ontogeny and homeostasis. Immunity. (2016) 44:439–49. 10.1016/j.immuni.2016.02.024 26982352

[3] Udalova IA Mantovani A Feldmann M. Macrophage heterogeneity in the context of rheumatoid arthritis. Nat Rev Rheumatol. (2016) 12:472–85. doi: 10.1038/nrrheum.2016.91 27383913

[4] Hoeffel G Florent G. Ontogeny of tissue-resident macrophages. Front Immunol. (2015) 6:486. doi: 10.3389/fimmu.2015.00486 26441990 PMC4585135

[5] Park MD Silvin A Ginhoux F Merad M. Macrophages in health and disease. Cell. (2022) 185:4259–79. 10.1016/j.cell.2022.10.007 PMC9908006 36368305

[6] Lazarov T Juarez-Carreño S Cox N Geissmann F. Physiology and diseases of tissue-resident macrophages. Nature. (2023) 618:698–707. 37344646 10.1038/s41586-023-06002-x PMC10649266

[7] Jacome-Galarza CE Percin GI Muller JT Mass E Lazarov T Eitler J. Developmental origin, functional maintenance and genetic rescue of osteoclasts. Nature. (2019) 568:541–5. doi: 10.1038/s41586-019-1105-7 PMC6910203 30971820

[8] Yahara Y Barrientos T Tang YJ Puviindran V Nadesan P Zhang H. Erythromyeloid progenitors give rise to a population of osteoclasts that contribute to bone homeostasis and repair. Nat Cell Biol. (2020) 22:49–59. doi: 10.1038/s41556-019-0437-8 31907410 PMC6953622

[9] Alivernini S MacDonald L Elmesmari A Finlay S Tolusso B Gigante MR. Distinct synovial tissue macrophage subsets regulate inflammation and remission in rheumatoid arthritis. Nat Med. (2020) 26:1295–306. doi: 10.1038/s41591-020-0939-8 32601335

[10] Thomas JR Appios A Calderbank EF Yoshida N Zhao X Hamilton RS. Primitive haematopoiesis in the human placenta gives rise to macrophages with epigenetically silenced HLA-DR. Nat Communications. (2023) 14:1764. 10.1038/s41467-023-37383-2 PMC10063560 36997537

[11] Gomez Perdiguero E Klapproth K Schulz C Busch K Azzoni E Crozet L. Tissue-resident macrophages originate from yolk-sac-derived erythro-myeloid progenitors. Nature. (2015) 518:547–51. 10.1038/nature13989 PMC5997177 25470051

[12] Mass E Ballesteros I Farlik M Halbritter F Günther P Crozet L. Specification of tissue-resident macrophages during organogenesis. Science. (2016) 353:aaf4238. doi: 10.1126/science.aaf4238 27492475 PMC5066309

[13] Bertrand JY Jalil A Ml K Jung S Cumano A Godin I. Three pathways to mature macrophages in the early mouse yolk sac. Blood. (2005) 106:3004–11. 10.1182/blood-2005-02-0461 16020514

[14] Palis J Robertson S Kennedy M Wall C Keller G. Development of erythroid and myeloid progenitors in the yolk sac and embryo proper of the mouse. Development. (1999) 126:5073–84. doi: 10.1242/dev.126.22.5073 10529424

[15] Ginhoux F Greter M Leboeuf M Nandi S See P Gokhan S. Fate mapping analysis reveals that adult microglia derive from primitive macrophages. Science. (2010) 330:841–5. 10.1126/science.1194637 PMC3719181 20966214

[16] Cumano A Godin I. Ontogeny of the hematopoietic system. Annu Rev Immunol. (2007) 25:745–85. doi: 10.1146/annurev.immunol.25.022106.141538 17201678

[17] Christensen JL Wright DE Wagers AJ Weissman IL. Circulation and chemotaxis of fetal hematopoietic stem cells. PloS Biol. (2004) 2:e75. doi: 10.1371/journal.pbio.0020075 15024423 PMC368169

[18] Zeng Y He J Bai Z Li Z Gong Y Liu C. Tracing the first hematopoietic stem cell generation in human embryo by single-cell RNA sequencing. Cell Res. (2019) 29:881–94. doi: 10.1038/s41422-019-0228-6 PMC6888893 31501518

[19] Popescu D-M Botting RA Stephenson E Green K Webb S Jardine L. Decoding human fetal liver haematopoiesis. Nature. (2019) 574:365–71. 10.1038/s41586-019-1652-y PMC6861135 31597962

[20] Jardine L Webb S Goh I Quiroga Londoño M Reynolds G Mather M. Blood and immune development in human fetal bone marrow and Down syndrome. Nature. (2021) 598:327–31. 10.1038/s41586-021-03929-x PMC7612688 34588693

[21] Patel AA Zhang Y Fullerton JN Boelen L Rongvaux A Maini AA. The fate and lifespan of human monocyte subsets in steady state and systemic inflammation. J Exp Med. (2017) 214:1913–23. doi: 10.1084/jem.20170355 PMC5502436 28606987

[22] Liu Z Gu Y Chakarov S Bleriot C Kwok I Chen X. Fate mapping via ms4a3-expression history traces monocyte-derived cells. Cell. (2019) 178:1509–25.e19. doi: 10.1016/j.cell.2019.08.009 31491389

[23] Hashimoto D Chow A Noizat C Teo P Beasley Mary B Leboeuf M. Tissue-resident macrophages self-maintain locally throughout adult life with minimal contribution from circulating monocytes. Immunity. (2013) 38:792–804. 23601688 10.1016/j.immuni.2013.04.004 PMC3853406

[24] Cytlak U Resteu A Pagan S Green K Milne P Maisuria S. Differential IRF8 transcription factor requirement defines two pathways of dendritic cell development in humans. Immunity. (2020) 53:353–70 e8. doi: 10.1016/j.immuni.2020.07.003 32735845 PMC7447982

[25] Guilliams M Lianne van de L. A hitchhiker’s guide to myeloid cell subsets: Practical implementation of a novel mononuclear phagocyte classification system. Front Immunol. (2015) 6:1-12. doi: 10.3389/fimmu.2015.00406 26322042 PMC4531301

[26] Hasegawa T Kikuta J Sudo T Matsuura Y Matsui T Simmons S. Identification of a novel arthritis-associated osteoclast precursor macrophage regulated by FoxM1. Nat Immunol. (2019) 20:1631–43. doi: 10.1038/s41590-019-0526-7 31740799

[27] Trzebanski S Kim J-S Larossi N Raanan A Kancheva D Bastos J. Classical monocyte ontogeny dictates their functions and fates as tissue macrophages. Immunity. (2024) 57:1225–42.e6. doi: 10.1016/j.immuni.2024.04.019 38749446

[28] Parsonage G Filer AD Haworth O Nash GB Rainger GE Salmon M. A stromal address code defined by fibroblasts. Trends Immunol. (2005) 26:150–6. doi: 10.1016/j.it.2004.11.014 PMC3121558 15745857

[29] McGettrick HM Butler LM Buckley CD Rainger GE Nash GB. Tissue stroma as a regulator of leukocyte recruitment in inflammation. J Leukoc Biol. (2012) 91:385–400. doi: 10.1189/jlb.0911458 22227966

[30] Buckley CD McGettrick HM. Leukocyte trafficking between stromal compartments: lessons from rheumatoid arthritis. Nat Rev Rheumatol. (2018) 14:476–87. doi: 10.1038/s41584-018-0042-4 30002464

[31] Scott CL Zheng F De Baetselier P Martens L Saeys Y De Prijck S. Bone marrow-derived monocytes give rise to self-renewing and fully differentiated Kupffer cells. Nat Communications. (2016) 7:10321. 10.1038/ncomms10321 PMC4737801 26813785

[32] Bruttger J Karram K Wörtge S Regen T Marini F Hoppmann N. Genetic cell ablation reveals clusters of local self-renewing microglia in the mammalian central nervous system. Immunity. (2015) 43:92–106. 26163371 10.1016/j.immuni.2015.06.012

[33] Kim J-S Trzebanski S Shin S-H Ilani NC Kaushansky N Scheller M. Monocyte-derived microglia with Dnmt3a mutation cause motor pathology in aging mice. bioRxiv. (2023) 2023:11.16.567402.

[34] Hasegawa T Kikuta J Sudo T Yamashita E Seno S Takeuchi T. Development of an intravital imaging system for the synovial tissue reveals the dynamics of CTLA-4 Ig *in vivo* . Sci Rep. (2020) 10:13480. doi: 10.1038/s41598-020-70488-y 32778803 PMC7417741

[35] Strobl J Gail LM Krecu L Madad S Kleissl L Unterluggauer L. Diverse macrophage populations contribute to distinct manifestations of human cutaneous graft-versus-host disease. Br J Dermatol. (2024) 190:402–14. doi: 10.1093/bjd/ljad402 PMC10873647 38010706

[36] Nayak DK Zhou F Xu M Huang J Tsuji M Hachem R. Long-term persistence of donor alveolar macrophages in human lung transplant recipients that influences donor-specific immune responses. Am J Transplantation. (2016) 16:2300–11. 10.1111/ajt.13819 PMC5289407 27062199

[37] Smith MD. The normal synovium. Open Rheumatol J. (2011) 5:100–6. doi: 10.2174/1874312901105010100 PMC3263506 22279508

[38] Buckley CD Ospelt C Gay S Midwood KS. Location, location, location: how the tissue microenvironment affects inflammation in RA. Nat Rev Rheumatol. (2021) 17:195–212. doi: 10.1038/s41584-020-00570-2 33526927

[39] Hasegawa T Lee CYC Hotchen AJ Fleming A Singh R Suzuki K. Macrophages and nociceptor neurons form a sentinel unit around fenestrated capillaries to defend the synovium from circulating immune challenge. Nat Immunol. (2024) 25:2270–83. doi: 10.1038/s41590-024-02011-8 PMC11588661 39587345

[40] Zhang B He P Lawrence JEG Wang S Tuck E Williams BA. A human embryonic limb cell atlas resolved in space and time. Nature. (2024) 635:668–78. doi: 10.1038/s41586-023-06806-x PMC7616500 38057666

[41] Kurowska-Stolarska M Alivernini S. Synovial tissue macrophages in joint homeostasis, rheumatoid arthritis and disease remission. Nat Rev Rheumatol. (2022) 18:384–97. doi: 10.1038/s41584-022-00790-8 35672464

[42] Zheng Y Wei K Jiang P Zhao J Shan Y Shi Y. Macrophage polarization in rheumatoid arthritis: signaling pathways, metabolic reprogramming, and crosstalk with synovial fibroblasts. Front Immunol. (2024) 15:1394108. doi: 10.3389/fimmu.2024.1394108 38799455 PMC11116671

[43] Culemann S Gruneboom A Nicolas-Avila JA Weidner D Lammle KF Rothe T. Locally renewing resident synovial macrophages provide a protective barrier for the joint. Nature. (2019) 572:670–5. 10.1038/s41586-019-1471-1 PMC6805223 31391580

[44] MacDonald L Elmesmari A Somma D Frew J Di Mario C Madhu R. Synovial tissue myeloid dendritic cell subsets exhibit distinct tissue-niche localization and function in health and rheumatoid arthritis. Immunity. (2024) 57:2843–62.e12. doi: 10.1016/j.immuni.2024.11.004 39609125

[45] Luse SA. A synovial sarcoma studied by electron microscopy. Cancer. (1960) 13:312–22. 10.1002/1097-0142(196003/04)13:2<312::aid-cncr2820130215>3.0.co;2-0 14419022

[46] Lever JD Ford EHR. Histological, histochemical and electron microscopic observations on synovial membrane. Anatomical Record. (1958) 132:525–39. 10.1002/ar.1091320402 13650183

[47] Barland P Novikoff AB Hamerman D. ELECTRON MICROSCOPY OF THE HUMAN SYNOVIAL MEMBRANE. J Cell Biol. (1962) 14:207–20. doi: 10.1083/jcb.14.2.207 PMC2106097 13865038

[48] Qu F Guilak F Robert LM. Cell migration: implications for repair and regeneration in joint disease. Nat Rev Rheumatol. (2019) 15:167–79. doi: 10.1038/s41584-018-0151-0 PMC7004411 30617265

[49] Tu J Hong W Guo Y Zhang P Fang Y Wang X. Ontogeny of synovial macrophages and the roles of synovial macrophages from different origins in arthritis. Front Immunol. (2019) 10. doi: 10.3389/fimmu.2019.01146 PMC6558408 31231364

[50] Sierro F Evrard M Rizzetto S Melino M Mitchell AJ Florido M. A liver capsular network of monocyte-derived macrophages restricts hepatic dissemination of intraperitoneal bacteria by neutrophil recruitment. Immunity. (2017) 47:374–88.e6. doi: 10.1016/j.immuni.2017.07.018 28813662

[51] Sun R Jiang H. Border-associated macrophages in the central nervous system. J Neuroinflammation. (2024) 21:67. 38481312 10.1186/s12974-024-03059-x PMC10938757

[52] Tsukasaki M Huynh NC-N Okamoto K Muro R Terashima A Kurikawa Y. Stepwise cell fate decision pathways during osteoclastogenesis at single-cell resolution. Nat Metab. (2020) 2:1382–90. doi: 10.1038/s42255-020-00318-y 33288951

[53] Riedlova P Sood S Goodyear CS Ansalone C. Differentiation of functional osteoclasts from human peripheral blood CD14+ Monocytes. JoVE. (2023) 191):e64698. doi: 10.3791/64698 36779608

[54] Omata Y Okada H Uebe S Izawa N Ekici AB Sarter K. Interspecies single-cell RNA-seq analysis reveals the novel trajectory of osteoclast differentiation and therapeutic targets. JBMR Plus. (2022) 6:e10631. doi: 10.1002/jbm4.10631 35866155 PMC9289986

[55] Mcdonald MM Kim SA Mulholland SB Rauner M. New insights into osteoclast biology. JBMR Plus. (2021) 5(9):e10539. doi: 10.1002/jbm4.10539 34532619 PMC8441501

[56] McDonald MM Khoo WH Ng PY Xiao Y Zamerli J Thatcher P. Osteoclasts recycle via osteomorphs during RANKL-stimulated bone resorption. Cell. (2021) 184:1330–47.e13. 33636130 10.1016/j.cell.2021.02.002 PMC7938889

[57] Ansalone C Cole J Chilaka S Sunzini F Sood S Robertson J. TNF is a homoeostatic regulator of distinct epigenetically primed human osteoclast precursors. Ann Rheumatic Diseases. (2021) 80:748. 10.1136/annrheumdis-2020-219262 PMC8142443 33692019

[58] Elson A Anuj A Barnea-Zohar M Reuven N. The origins and formation of bone-resorbing osteoclasts. Bone. (2022) 164:116538. doi: 10.1016/j.bone.2022.116538 36028118

[59] Feil S Krauss J Thunemann M Feil R. Genetic inducible fate mapping in Adult mice using tamoxifen-dependent cre recombinases. In: Singh SR Coppola V, editors. Mouse genetics: methods and protocols. Springer New York, New York, NY (2014). p. 113–39. 10.1007/978-1-4939-1215-5_6 25064100

[60] Zhang F Jonsson AH Nathan A Millard N Curtis M Xiao Q. Deconstruction of rheumatoid arthritis synovium defines inflammatory subtypes. Nature. (2023) 623:616–24. 10.1038/s41586-023-06708-y PMC10651487 37938773

[61] Zhang F Wei K Slowikowski K Fonseka CY Rao DA Kelly S. Defining inflammatory cell states in rheumatoid arthritis joint synovial tissues by integrating single-cell transcriptomics and mass cytometry. Nat Immunol. (2019) 20:928–42. doi: 10.1038/s41590-019-0378-1 PMC6602051 31061532

[62] van Hemert FJ Thurlings R Dohmen SE Voermans C Tak PP van Eck-Smit BL. Labeling of autologous monocytes with 99mTc-HMPAO at very high specific radioactivity. Nucl Med Biol. (2007) 34:933–8. doi: 10.1016/j.nucmedbio.2007.07.008 17998095

[63] Thurlings RM Wijbrandts CA Bennink RJ Dohmen SE Voermans C Wouters D. Monocyte scintigraphy in rheumatoid arthritis: the dynamics of monocyte migration in immune-mediated inflammatory disease. PloS One. (2009) 4:e7865. doi: 10.1371/journal.pone.0007865 19924229 PMC2773010

[64] Bennink RJ Thurlings RM van Hemert FJ Voermans C Dohmen SE van Eck-Smit BL. Biodistribution and radiation dosimetry of 99mTc-HMPAO-labeled monocytes in patients with rheumatoid arthritis. J Nucl Med. (2008) 49:1380–5. doi: 10.2967/jnumed.108.051755 18632808

[65] Lubin R Patel AA Mackerodt J Zhang Y Gvili R Mulder K. The lifespan and kinetics of human dendritic cell subsets and their precursors in health and inflammation. J Exp Medicine. (2024) 221:e20220867. doi: 10.1084/jem.20220867 PMC11488382 39417994

