# Supplementary material for: The ontogeny of synovial tissue macrophages
Source: Front Immunol. 2025 May 20;16:1603473. doi: 10.3389/fimmu.2025.1603473 (PMC12129757; doi:10.3389/fimmu.2025.1603473)
Supplement: Supplementary Table 1 — A taxonomy of human and mouse STM. *The MerTK+LYVE1+FOLR2high cluster was reported to be localised in the lining layer in both healthy and remission RA (9). [file Table1.docx]

|  | Tissue resident macrophages | | | | | Tissue infiltrating macrophages | | | |
| --- | --- | --- | --- | --- | --- | --- | --- | --- | --- |
| Human STM | TREM2^+^  (LYVE1^+^*) | LYVE1^+^ | ID2^+^ | | ICAM1^+^ | CD48^+^  S100A12^+^ | CD48^+^  SPP1^+^ | CD48^+^  ISG15^+^ | CD48^+^  HLA^high^  CLEC10A^+^ |
| Mouse STM | CX3CR1^+^  VSIG4^+^  LYVE1^+^ | MHC II^+^ M-CSFR^+^ RELMα^+^ | | | | CCR2^+^  IL-1β^+^ | CCR2^+^  IL-1β^+^ | CCR2^+^  ARG1^+^ | MHC II^high^  AQP1^+^ |
|  |  | CD11c^-^ | | CD11c^+^* | |  |  |  |  |
|  | Lining layer | Sub-lining layer | | | |  | | | |
